# Quarantine and demographic characteristics as predictors of perceived stress and stress responses during the third year of COVID-19 in China

**DOI:** 10.3389/fpsyt.2022.962285

**Published:** 2022-09-08

**Authors:** Qi Gao, Huijing Xu, Kaitian Shi, Yi Zhang, Cheng Zhang, Qian Jiang, Xiaoliang Wei, Taosheng Liu

**Affiliations:** ^1^Department of Psychiatry, Faculty of Psychology, Naval Medical University, Shanghai, China; ^2^Department of Political Theory, Qingdao Branch of Naval Aeronautical University, Qingdao, China; ^3^Department of Medical Psychology, Changzheng Hospital, Naval Medical University, Shanghai, China

**Keywords:** COVID-19, quarantine, perceived stress, stress responses, emotional response, physical response, behavioral response

## Abstract

**Background:**

Quarantine as one of the most effective epidemic prevention measures, significantly increased people's stress levels. Ongoing monitoring of the stress status of people under quarantine during the pandemic is an important part of assessing the long-term impact of COVID-19 on mental health. This study aimed to gain a more comprehensive understanding of the stress status of people under quarantine, including perceived stress and stress responses, during the third year of the COVID-19 pandemic in China.

**Methods:**

An anonymous online survey was conducted among 464 participants from 39 cities in China from March 31 to April 12, 2022. The survey included three questionnaires: a self-designed questionnaire collecting demographic information and quarantine characteristics, the Perceived Stress Scale (PSS-10) and the Stress Response Questionnaire (SRQ). The *t*-test or one-way ANOVA or the Welch *F*-test were used to examine the differences among demographic and quarantine variables of perceived stress and stress responses, then multiple linear regressions were performed to identify the predictors of perceived stress and stress responses.

**Results:**

428 valid respondents were finally included. The average scores of perceived stress, total stress response, emotional response, physical response, and behavioral response were 14.70 ± 7.02, 50.24 ± 22.48, 20.35 ± 9.99, 15.23 ± 7.25, and 11.39 ± 5.27, respectively. The regression analysis showed that the degree of financial worries and days of continuous quarantine were the predictors of perceived stress. The degree of financial worries was a vital factor in predicting total stress response, emotional response, physical response and behavioral response, and in predicting emotional response, age was also a significant predictor.

**Conclusion:**

The stress status of individuals under quarantine was generally stable but still needs further attention during the third year of the COVID-19 pandemic. People who are young, have a high degree of financial worries and have been quarantined for a long time may be at a higher risk of perceived stress and stress responses. Relevant authorities should pay closer attention to the risk groups, and additional support and assistance might be required for those mostly worried about their financial situations under quarantine.

## Introduction

Three years have passed since the outbreak of COVID-19, during which time the pandemic has continued to circulate widely and intensively around the world, with significant negative impacts on people's physical and mental health globally (1–4). The eleventh meeting of the Emergency Committee convened by the WHO declared that SARS-CoV-2 had not yet established its ecological niche and continued to lead to high levels of morbidity and mortality, particularly among vulnerable populations. The situation is more complicated than at it started (5). Therefore, WHO emphasized that public health and social measures (PHSM), including non-pharmaceutical individual and societal interventions (5), must continue to be maintained to limit the transmission of COVID-19 and reduce deaths. China is the country that has experienced the pandemic for the longest time and still adheres to rigorous quarantine measures (6). The psychological state of the Chinese people requires ongoing monitoring.

Quarantine has been proved to be one of the non-pharmacological interventions most strongly associated with significant negative psychological effects, both immediate and prolonged, such as high levels of stress, depression, anxiety, poor sleep, higher consumption of alcohol and tobacco, unhealthy diet behaviors, and even the development of mental disorders (7–12). However, there is no consensus on the effect of quarantine duration on mental health. Most studies suggested that the longer the quarantine, the poorer the individual's mental health (13–15). Other studies found that mental health problems showed inverted *U*-shaped changes, which increased rapidly in the early stages of lockdown and then gradually decreased (16–18). While research conducted by Wang et al. (19) reported that the levels of stress, anxiety and depression did not change significantly during the 4 weeks of quarantine. By contrast, some other studies yielded more mixed results. For example, a study of sentiment analysis on social media data discovered a *U*-shaped change in psychological problems, which was highest at the beginning of quarantine, then decreased, and was lowest around the 13th day, after which the psychological problems increased again (20). Another study conducted in Spain observed heterogeneous changes in mental health problems across quarantine duration, with a significant increase in depression, no significant change in anxiety, and a clear downward trend in PTSD (21). Overall, the effects of quarantine duration on mental health presented a complex pattern. China has implemented precise and differentiated epidemic control strategies based on different conditions in each region. The places where people are quarantined and the scope of their activities vary depending on the risk of epidemic transmission in the area where the outbreak occurred (22). The epidemic areas can be classified into three types: locked-down areas, controlled areas and precautionary areas (23). Locked-down areas are the residential or surrounding areas where confirmed and asymptomatic cases live or frequently engage in activities. Residents in locked-down areas are not permitted to leave their homes; Controlled areas are the areas where confirmed or asymptomatic cases have traveled. The time node spans 2 days before the onset of confirmed cases or asymptomatic cases testing positive to the time of being isolated. People in controlled areas are not allowed to leave the demarcated areas. Both locked-down areas and controlled areas can be accurately demarcated to communities, buildings, units, and so on. Precautionary areas refer to the areas where the epidemic occurs except the locked-down and controlled areas, and residents there are required not to cross counties or districts. Fewer studies have explored the effects of different quarantine locations and permitted mobility scopes on mental health, which means such effects are still unclear.

A recent review summarized the prevalence of various mental health problems during this pandemic, concluding that stress was the most common psychological problem among general public (24). As an important psychological symptom, stress is not only closely related to psychological illnesses such as anxiety and depression, but chronic stress can be detrimental to physiology, like suppressing the immune system and raising the risk of viral infection (25, 26). Lazarus emphasized that it was the perceptions of one's own stressfulness, not the objective stressful events, to some extent, that determined one's response to stressors (27). Perceived stress is the assessment of the degree to which the situation in one's life is perceived as stressful (28). And stress responses refers to the emotional, physical, and behavioral changes that individuals exhibit under the influence of stress (29). However, most previous studies on individual stress states during the COVID-19 pandemic only focused on perceived stress or did not distinguish between perceived stress and stress responses (2, 24, 30). The Perceived Stress Scale (PSS) and the stress subscale of the Depression Anxiety Stress Scales-21 (DASS-21) are two of most widely used scales for measuring stress in the topic of mental health under COVID-19 (31–33), while the former addresses only the perceived stress of individuals that to which degree they felt their lives unexpected, uncontrolled, and overloaded (34), and the latter mainly reflects overall stress characteristics such as tension, overreaction and irritability, without distinguishing between different stress components (35). Thus, a separate investigation of perceived stress and stress responses is urgently required in order to present a more comprehensive picture of individual stress characteristics under the COVID-19 pandemic.

Therefore, with the future of the pandemic still uncertain and quarantine measures likely to continue for a considerable period of time, the present study aimed to investigate the perceived stress and stress responses of the public under different quarantine conditions and to explore the particular susceptible population subgroups for stress throughout the quarantine period. In order to provide policymakers with scientific evidence to improve mental health services and reduce the potential psychological trauma of quarantined populations.

## Materials and methods

### Procedure and participants

An anonymous online survey was conducted from March 31 to April 12, 2022 by Questionnaire Star (www.wjx.cn), using convenience sampling and snowball sampling. The sample size was calculated by G^*^Power version 3.1.9.7 (36). *F*-test (Linear multiple regression: Fixed model, *R*^2^ increase) was selected and the minimum sample size was 179 individuals [Effect size = 0.15, α= 0.05, 1 – β = 0.1, Total number of predictors = 17 (at most 17 dummy variables).]. Finally, a total of 464 respondents were collected, of which 428 were valid (valid ratio = 92.24%, *M* = 35.44, *SD* = 11.05). The inclusion criteria included participants being 18 years of age or older, being quarantined, and voluntarily participating in the investigation. The exclusion criteria included response times of <2 min or more than 30 min, logical confusion, repeat answers, and subgroups with <10 participants. The valid participants were from 39 cities in 18 provinces in China.

The study was approved by the Ethics Committee of Naval Medical University. Before beginning the questionnaire, the participants were required to complete the informed consent forms, and were informed of the voluntary and confidential nature of their participation, as well as their ability to withdraw from the survey at any time.

### Instruments

#### Demographic information

Demographic variables included gender (Male, Female), age (18–35, 36–50, >50), occupations (Occupations have the risk of COVID-19 exposure: healthcare works, police, administrators whose work is directly involved with the pandemic, pandemic volunteers, etc.; Occupations have no risk of COVID-19 exposure: teachers, students, enterprise worker, freelancers, retires, etc.), the degree of financial worries [Extreme worry (Ext), Serious worry (Ser), Moderate worry (Mod), Mild worry (Mil), Not at all (NA).], quarantine locations (At home, At workplace, At dormitory), the permitted scope of movement [Not permitted to leave homes (NLH), Not permitted to leave buildings (NLB), Not permitted to leave communities or campus (NLCC), Not permitted to leave counties or districts (NLCD).], and days of continuous quarantine (0–7, 8–14, 15–21, 22–30, >30 days).

#### The perceived stress scale (PSS-10)

The Perceived Stress Scale is used to measure the degree of stress people perceived in the past month, including 10 items rated from 0 (never) to 4 (always). Four of the items are scored in reverse, and the total scores ranged from 0 to 40. Higher scores indicate higher perceived stress. The Cronbach's α of a Chinese version was 0.91 (37).

#### The stress response questionnaire (SRQ)

The Stress Response Questionnaire was first designed by Jiang (29) in order to assess corresponding psychosomatic symptoms and the degree of individual stress responses, including 28 items rated from 1 (surely yes) to 5 (surely not). SRQ consists of three subscales: Emotional Response (items 3, 5, 6, 9, 10, 14, 18, 21, 24, 25, 27, and 28), Physical Response (items 1, 4, 13, 15, 19, 20, 22, and 23), and Behavioral Response (items 7, 8, 11, 12, 17, and 26). The total stress response score is the sum of the three subscales' scores plus items 2 and 6. Higher scores indicate higher stress responses. The questionnaire has high credibility with the Cronbach's α of 0.90.

### Data analysis

Descriptive statistics were conducted to describe categorical variables with frequency (*n*) and percentages (%) and continuous variables with means (*M*) and standard deviations (*SD*). The Kolmogorov–Smirnov test and the rule of thumb for normality testing (38) were used to assess data normality. The independent sample *t*-test or one-way ANOVA was used to examine differences in perceived stress, total stress response, emotional response, physical response, and behavioral response among different demographic variables. The Welch *F*-test was used for variables that didn't satisfy the homogeneity of variance assumption. Then, several multiple linear regressions were performed to predict perceived stress, total stress response and other three stress responses based on those demographic variables that were significantly associated with the dependent variables (Normal P–P plots were used to verify data normality of residual variance before regressions.). All statistical tests were two-tailed with *p* < 0.05 as statistically significant. All data were analysed by SPSS21.0 (IBM SPSS Statistics for Windows, Version 21.0. Armonk, NY: IBM Corp).

## Results

### Sample characteristics

The demographic characteristics of all participants were shown in Table 1. 60.98% were female. About half of the participants were aged between 18 and 35 (53.74%). 19.86% had occupations under the risk of COVID-19 exposure. 6.78% of the participants reported they were extremely worried about their financial situations during the COVID-19 pandemic, while 26.87% expressed no worry at all. More than two-third of the participants were quarantined at home (64.49%). Nearly one-fourth of the samples were not allowed to leave their homes during the quarantine period (26.17%). 35.05% had been continuously quarantined for 0–7 days, and 7.24% for already more than 30 days.

**Table 1:** Univariate analysis of factors associated with perceived stress, total stress response, emotional response, physical stress, and behavioral stress.

		**Perceived stress**		**Total stress response**		**Emotional response**		**Physical response**		**Behavioral response**	
	***n* (%)**	***M* ±*SD***	***t*/*F***	***M* ±*SD***	***t*/*F***	***M* ±*SD***	***t*/*F***	***M* ±*SD***	***t*/*F***	***M* ±*SD***	***t*/*F***
**Gender**			−2.36*		0.30		−0.22		−0.29		0.99
Male	167 (39.02)	13.71 ± 7.44		50.65 ± 23.59		20.23 ± 10.47		15.10 ± 7.23		11.71 ± 5.62	
Female	261 (60.98)	15.34 ± 6.68		49.98 ± 21.78		20.44 ± 9.68		15.31 ± 7.28		11.19 ± 5.03	
**Age**			7.24***		4.70^Δ*^		7.30^Δ**^		3.55^Δ*^		2.59
18–35	230 (53.74)	15.85 ± 7.10		53.24 ± 23.64		22.00 ± 10.50		16.08 ± 7.60		11.88 ± 5.53	
36–50	163 (38.08)	13.57 ± 6.74		47.07 ± 20.73		18.61 ± 9.17		14.29 ± 6.57		10.93 ± 4.99	
>50	35 (8.18)	12.43 ± 6.50		45.31 ± 19.98		17.68 ± 8.36		13.94 ± 7.34		10.31 ± 4.42	
**Occupations**			1.21		0.73		0.53		0.80		0.80
Have the risk of COVID-19 exposure	85 (19.86)	15.53 ± 6.85		51.84 ± 22.60		20.87 ± 10.37		15.79 ± 7.29		11.80 ± 5.01	
Have no risk of COVID-19 exposure	343 (80.14)	14.50 ± 7.06		49.85 ± 22.46		20.23 ± 9.90		15.09 ± 7.24		11.29 ± 5.33	
**The degree of financial worries**			23.49***		10.90^Δ***^		12.15^Δ***^		8.05^Δ***^		10.85^***Δ^
Extreme worry	29 (6.78)	20.72 ± 8.91		67.66 ± 35.57		28.07 ± 15.71		20.03 ± 10.87		15.45 ± 8.03	
Serious worry	70 (16.36)	18.06 ± 6.17		58.36 ± 24.93		24.21 ± 11.39		17.19 ± 7.63		13.53 ± 5.65	
Moderate worry	136 (31.78)	15.80 ± 6.37		51.82 ± 20.07		21.14 ± 8.81		15.90 ± 6.83		11.51 ± 4.90	
Mild worry	78 (18.22)	13.06 ± 5.86		46.36 ± 19.84		18.58 ± 8.75		14.18 ± 6.51		10.64 ± 4.68	
Not at all	115 (26.87)	10.96 ± 6.11		41.67 ± 16.04		16.36 ± 6.89		12.73 ± 5.73		9.43 ± 3.77	
**Quarantine locations**			1.32		0.48		0.94		0.38		0.24
At home	276 (64.49)	14.37 ± 6.87		49.90 ± 22.35		20.09 ± 9.92		15.07 ± 7.12		11.44 ± 5.31	
At workplace	54 (12.62)	14.59 ± 7.50		48.67 ± 21.56		19.62 ± 9.51		15.00 ± 7.05		10.93 ± 5.31	
At dormitory	98 (22.90)	15.70 ± 7.15		52.06 ± 23.44		21.54 ± 10.44		15.79 ± 7.75		11.50 ± 5.16	
**The permitted scope of movement**			4.25**		2.48		2.74^Δ*^		2.84^Δ*^		1.55
Not permitted to leave homes	112 (26.17)	12.96 ± 6.40		46.70 ± 18.89		18.54 ± 8.18		13.95 ± 5.96		11.00 ± 4.69	
Not permitted to leave buildings	128 (29.91)	16.16 ± 7.40		54.53 ± 25.19		22.13 ± 11.13		16.70 ± 8.44		12.28 ± 5.83	
Not permitted to leave communities or campus	168 (39.25)	14.70 ± 6.95		49.42 ± 21.62		20.19 ± 9.72		15.01 ± 6.85		10.99 ± 5.04	
Not permitted to leave counties or districts	20 (4.67)	15.10 ± 6.79		49.55 ± 26.75		20.60 ± 12.29		14.80 ± 7.75		11.25 ± 6.03	
**Days of continuous quarantine**			5.78***		2.82^Δ*^		3.26^Δ*^		2.62*		1.56
0–7 days	150 (35.05)	12.71 ± 6.15		45.87 ± 19.93		18.26 ± 8.62		13.93 ± 6.58		10.67 ± 4.76	
8–14 days	53 (12.38)	14.15 ± 7.05		48.85 ± 20.17		19.89 ± 9.21		14.43 ± 6.62		11.25 ± 4.82	
15–21 days	105 (24.53)	16.16 ± 7.77		54.03 ± 23.58		21.56 ± 10.44		16.50 ± 7.76		12.32 ± 5.52	
22–30 days	89 (20.79)	16.06 ± 6.74		52.64 ± 25.13		21.89 ± 11.25		16.02 ± 7.59		11.54 ± 6.02	
>30 days	31 (7.24)	16.45 ± 6.95		54.03 ± 23.52		22.87 ± 10.67		16.23 ± 7.83		11.39 ± 5.27	

M, mean; SD, standard deviation.

^Δ^The Welch F test.

^*^p < 0.05, ^**^p < 0.01, ^***^p < 0.001.

### Differences of demographic variables in perceived stress

Table 1 also included the *t*-test or ANOVA results on perceived stress scores among demographic variables. The average score of perceived stress was 14.70 ± 7.02. Females reported significantly higher perceived stress than males. Participants aged 18–35 had significantly higher perceived stress scores than those aged 36–50 and over 50. Participants belonging to Ext and Ser reported significantly higher perceived stress than Mod, Mil, and NA. Participants in Mod reported higher perceived stress than Mil and NA, and those in Mil group had higher perceived stress than NA. In the aspect of permitted scope of movement, respondents who were NLH reported the lowest levels of perceived stress, significantly lower than those who were NLB, NLCC, and NLCD. In days of continuous quarantine, participants who were quarantined for 0–7 days had significantly lower levels of perceived stress than those who were quarantined for more than 15 days. Interestingly, the average perceived stress score of participants quarantined for 15–21 days was higher than that of participants quarantined for 22–30 days, and the average increased again after 30 days. Occupations and quarantine locations did not show any significant differences.

### Differences of demographic variables in total stress response, emotional response, physical response, and behavioral response

The results were presented in Table 1.

The average score of total stress response was 50.24 ± 22.48. The total stress response was significantly higher in the 18–35 age group than in the 36–50 age group. Participants in Ext and Ser reported higher total stress responses than those in Mil and NA, and those in Mod than NA. The total stress response of participants quarantined for 0–7 days was significantly lower than that for 15–21 days. There was no significant difference in other demographic variables.

In terms of the emotional response, the average score of emotional response was 20.35 ± 9.99. Compared with the 36–50 and over 50 age groups, young people aged 18–35 years old reported higher emotional responses. Respondents in Ext and Ser had higher emotional responses than those in Mil and NA, and those in Mod than NA. Participants in NLH reported lower emotional responses than in NLB. And in days of continuous quarantine, the average score of emotional response reported between 0 and 7 days was the lowest. There were no significant differences in emotional response by gender, occupations, or quarantine locations.

In terms of the physical response, the average score of physical response was 15.23 ± 7.25. Participants aged 18–35 reported higher physical responses than those aged 35–50. Participants in Ext, Ser, and Mod reported higher physical responses than those in NA. Participants in NLH reported lower physical responses in contrast with NLB. The physical response of participants quarantined for 0–7 days was lower than that for 15–21 and 21–30 days. There was no significant difference in other demographic variables.

The average score of behavioral response was 11.39 ± 5.27, only the degree of financial worries differed significantly, participants in Ext and Ser had higher behavioral responses than those in Mil and NA, and those in Mod than NA.

### Predictors of perceived stress, total stress response, emotional response, physical response, and behavioral response

The significant demographic variables in Table 1 were included into multiple linear regressions to obtain predictors of perceived stress, total stress response, emotional response, physical response, and behavioral response, respectively. The results were displayed in Tables 2–**6**.

**Table 2:** Multiple linear regression analysis on perceived stress.

	**β**	**SE**	**Beta**	***t***	***p***	**95%CI**
**Constant**	17.43	1.77		9.85	<0.001***	[13.95, 20.92]
**Gender**	1.12	0.64	0.08	1.77	0.08	[−0.12, 2.38]
**Age (Ref: 18–35)**						
36–50	−0.99	0.75	−0.07	−1.31	0.19	[−2.47, 0.05]
>50	−0.72	1.23	−0.03	−0.59	0.56	[−3.13, 1.69]
**The degree of financial worries (Ref: Not at all)**						
Extreme worry	9.34	1.33	0.34	7.04	<0.001***	[6.74, 11.95]
Serious worry	6.71	0.96	0.35	6.97	<0.001***	[4.82, 8.60]
Moderate worry	4.62	0.80	0.31	5.76	<0.001***	[3.04, 6.19]
Mild worry	2.25	0.92	0.12	2.43	0.02**	[0.43, 4.07]
**The permitted scope of movement (Ref: Not permitted to leave homes)**						
Not permitted to leave buildings	0.98	0.95	0.06	1.03	0.30	[−0.88, 2.83]
Not permitted to leave communities or campus	−0.03	0.96	0.00	−0.03	0.98	[−1.92, 1.87]
Not permitted to leave counties or districts	0.84	1.61	0.03	0.52	0.60	[−2.32, 4.00]
**Days of continuous quarantine (Ref: 0–7 days)**						
8–14 days	1.43	1.11	0.07	1.29	0.20	[−0.75, 3.61]
15–21 days	2.41	0.99	0.15	2.43	0.02**	[0.46, 4.35]
22–30 days	1.86	1.03	0.11	1.81	0.07	[−0.16, 3.88]
>30 days	2.19	1.47	0.08	1.49	0.14	[−0.70, 5.08]
***R* ^2^**	0.21					
***F***	8.93					
***p***	<0.001***					

^**^p < 0.01, ^***^p < 0.001. β,Unstandardized coefficients, Beta, Standardized coefficients, 95%CI, 95% confidence intervals.

The regression results with perceived stress as the dependent variable showed that the degree of financial worries and days of continuous quarantine were the predictors of perceived stress (Table 2). To be more specific, Ext, Ser, Mod, and Mil all predicted higher perceived stress than NA. Compared with being continuously quarantined for 0–7 days, being quarantined for 15–21 days predicted higher perceived stress. The linear regression model explained 21.00% of the variance in perceived stress (*R*^2^ = 0.21, *F* = 8.93).

As the regression analysis shown in Tables 3–6, the linear regression models explained 11.00 (*R*^2^ = 0.11, *F* = 6.27), 12.00 (*R*^2^ = 0.12, *F* = 5.59), 8.00 (*R*^2^ = 0.08, *F* = 3.87), and 10.00% (*R*^2^ = 0.10, *F* = 12.85) of the variance in the total stress response, emotional response, physical response, and behavioral response, respectively. The degree of financial worries was a predictor of all the stress responses. Specifically, Ext, Ser, and Mod predicted higher total stress responses, emotional responses, physical responses, and behavioral responses than NA. Age was only a predictor of emotional response, the middle-aged group (36–50) was less likely to report higher emotional responses when compared with the younger group (18–35).

**Table 3:** Multiple linear regression analysis on total stress response.

	**β**	**SE**	**Beta**	***t***	***p***	**95%CI**
**Constant**	66.73	4.85		13.77	<0.001***	[57.21, 76.26]
**Age (Ref:18–35)**						
36–50	−4.43	2.51	−0.10	−1.76	0.08	[−9.37, 0.51]
>50	−2.84	4.08	−0.04	−0.70	0.49	[−10.87, 5.19]
**The degree of financial worries (Ref: Not at all)**						
Extreme worry	25.33	4.48	0.28	5.66	<0.001***	[16.53, 34.14]
Serious worry	16.43	3.24	0.27	5.07	<0.001***	[10.05, 22.80]
Moderate worry	10.08	2.70	0.21	3.74	<0.001***	[4.78, 15.38]
Mild worry	5.01	3.13	0.09	1.60	0.11	[−1.14, 11.15]
**Days of continuous quarantine (Ref: 0–7 days)**						
8–14 days	2.47	3.51	0.04	0.71	0.48	[−4.43, 9.37]
15–21 days	4.95	2.98	0.10	1.66	0.10	[−0.91, 10.80]
22–30 days	2.63	3.17	0.05	0.83	0.41	[−3.60, 8.86]
>30 days	2.41	4.56	0.03	0.53	0.60	[−6.56, 11.37]
***R* ^2^**	0.11					
***F***	6.27					
***p***	<0.001***					

^***^p < 0.001. β,Unstandardized coefficients, Beta, Standardized coefficients, 95%CI, 95% confidence intervals.

**Table 4:** Multiple linear regression analysis on emotional response.

	**β**	**SE**	**Beta**	***t***	***p***	**95%CI**
**Constant**	27.67	2.21		12.50	<0.001***	[23.32, 32.02]
**Age (Ref:18–35)**						
36–50	−2.42	1.13	−0.12	−2.15	0.03**	[−4.63, −0.20]
>50	−2.04	1.82	−0.06	−1.12	0.26	[−5.62, 1.54]
**The degree of financial worries (Ref: Not at all)**						
Extreme worry	11.25	1.98	0.28	5.68	<0.001***	[−7.85, 0.36]
Serious worry	7.51	1.44	0.28	5.23	<0.001***	[−10.50, −2.88]
Moderate worry	4.56	1.20	0.21	3.81	<0.001***	[−12.89, −4.70]
Mild worry	2.46	1.38	0.10	1.78	0.08	[−15.15, −7.36]
**The permitted scope of movement (Ref:Not permitted to leave homes)**						
Not permitted to leave buildings	1.02	1.41	0.05	0.73	0.47	[−1.75, 3.80]
Not permitted to leave communities or campus	−0.57	1.44	−0.03	−0.40	0.69	[−3.40, 2.25]
Not permitted to leave counties or districts	0.42	2.40	0.01	0.17	0.86	[−4.30, 5.13]
**Days of continuous quarantine (Ref: 0–7 days)**						
8–14 days	1.32	1.66	0.04	0.79	0.43	[−1.95, 4.58]
15–21 days	1.73	1.48	0.08	1.17	0.24	[−1.18, 4.63]
22–30 days	1.44	1.54	0.06	0.94	0.35	[−1.58, 4.46]
>30 days	2.19	2.20	0.06	1.00	0.32	[−2.13, 6.52]
***R* ^2^**	0.12					
***F***	5.59					
***p***	<0.001***					

^**^p < 0.01, ^***^p < 0.001. β,Unstandardized coefficients, Beta, Standardized coefficients, 95%CI, 95% confidence intervals.

**Table 5:** Multiple linear regression analysis on physical response.

	**β**	**SE**	**Beta**	***t***	***p***	**95%CI**
**Constant**	19.07	1.65		11.59	<0.001***	[15.84, 22.30]
**Age (Ref:18–35)**						
36–50	−0.97	0.84	−0.07	−1.16	0.25	[−2.62, 0.68]
>50	−0.47	1.35	−0.02	−0.35	0.73	[−3.13, 2.19]
**The degree of financial worries (Ref: Not at all)**						
Extreme worry	6.91	1.47	0.24	4.69	<0.001***	[4.02, 9.80]
Serious worry	4.25	1.07	0.22	3.98	<0.001***	[2.15, 6.34]
Moderate worry	2.97	0.89	0.19	3.34	<0.001***	[1.23, 4.72]
Mild worry	1.57	1.03	0.08	1.53	0.13	[−0.45, 3.59]
**The permitted scope of movement (Ref: Not permitted to leave homes)**						
Not permitted to leave buildings	1.34	1.05	0.09	1.28	0.20	[−0.73, 3.40]
Not permitted to leave communities or campus	−0.15	1.07	−0.01	−0.14	0.89	[−2.25, 1.95]
Not permitted to leave counties or districts	−0.11	1.78	0.00	−0.06	0.95	[−3.62, 3.39]
**Days of continuous quarantine (Ref: 0–7 days)**						
8–14 days	0.42	1.23	0.02	0.34	0.73	[−2.01, 2.85]
15–21 days	1.74	1.10	0.10	1.59	0.11	[−0.42, 3.90]
22–30 days	0.87	1.14	0.05	0.76	0.45	[−1.38, 3.11]
>30 days	1.17	1.64	0.04	0.72	0.47	[−2.04, 4.39]
***R* ^2^**	0.08					
***F***	3.87					
***p***	<0.001***					

^***^p < 0.001. β,Unstandardized coefficients, Beta, Standardized coefficients, 95%CI, 95% confidence intervals.

**Table 6:** Multiple linear regression analysis on behavioral response.

	**β**	**SE**	**Beta**	***t***	***p***	**95%CI**
**Constant**	15.45	0.93		16.65	<0.001***	[13.63, 17.27]
**The degree of financial worries (Ref: Not at all)**						
Extreme worry	6.01	1.04	0.29	5.79	<0.001***	[3.97, 8.05]
Serious worry	4.09	0.76	0.29	5.41	<0.001***	[2.61, 5.58]
Moderate worry	2.07	0.63	0.18	3.28	<0.001***	[0.83, 3.32]
Mild worry	1.21	0.73	0.09	1.65	0.10	[−0.23, 2.65]
***R* ^2^**	0.10					
***F***	12.85					
***p***	<0.001***					

^***^p < 0.001. β,Unstandardized coefficients, Beta, Standardized coefficients, 95%CI, 95% confidence intervals.

## Discussion

This study examined the perceived stress and stress responses of individuals with different quarantine conditions and demographic characteristics during the third year of the COVID-19 pandemic in China, and draw some meaningful conclusions. The average perceived stress score of quarantined individuals was 14.70 ± 7.02, lower than the result of Gamonal Limcaoco's study of public perceived stress in 41 countries using the same scale in the early stage of the pandemic (17.40 ± 6.40) (39) and also lower than the perceived stress score of quarantined people investigated by Dale et al. (40) during the second wave of the pandemic at a later time period (16.42 ± 7.6). In terms of stress responses, the average scores of the total stress response (50.24 ± 22.48), emotional response (20.35 ± 9.99), physical response (15.23 ± 7.25), and behavioral response (11.39 ± 5.27) were all far lower than the results of Wan's study in the initial stage of the pandemic (61.10 ± 27.30, 24.90 ± 12.60, 19.00 ± 8.40, 13.40 ± 6.20) (41). This may reflects an adaptive pattern of mental states when people are exposed to stressful or traumatic events that mental health problems will increase sharply at first, then gradually decrease through adaption and adjustment as time passes (4, 18, 42, 43). Also, it can be inferred that with improved public understanding of COVID-19, increased vaccination coverage, the development of specific medicine, as well as the normalization of epidemic prevention and control (44), people's perceived stress and stress responses were both lower than in previous studies, even though they were in quarantine.

Age was found to be a predictor of emotional response, with younger people having higher emotional responses. Previous studies have already confirmed the vulnerability of young people to stress during the pandemic (14, 45, 46), which may be due to their greater social, academic, work, and economic challenges and their higher consumption of pandemic news *via* social media (14, 45, 46). Additionally, it was also found that older people were more resilient than younger ones (48), allowing them to deal with the pandemic and quarantine more calmly.

The degree of financial worries was a key predictor of all dependent variables. The higher the level of financial worries, the more likely individuals were to suffer higher perceived stress and stress responses. Many researches have proved the financial factor as one of the main predictors of psychological stress during the pandemic (14, 49–51). Quarantined people are directly in danger of job suspension or unemployment, resulting in decreased or, in the worst scenario, no income, which certainly contributes to people's stress levels rising. More importantly, these kind of effects could be long-lasting even after the pandemic (49).

In quarantine characteristics, days of continuous quarantine were associated with perceived stress, and 15–21 days of quarantine was a risk factor for predicting perceived stress. The variation trend of the average perceived stress in quarantine duration reflected some interesting results: the average perceived stress during 0–7 days was the lowest, then gradually increased, peaking during 15–21 days, slightly decreasing during 22–30 days, and then increasing again after 30 days. On the whole, our results were consistent with previous studies concluding that people's perceived stress would increase as quarantine time extended (13–15). However, perceived stress did not rise in a straight line but rather fluctuated. It should be noted, in particular, that people's perceived stress peaked during the 15–21 days of quarantine. Special attention should be paid to the individuals' mental health at this time. The total and physical stress responses followed the same pattern as perceived stress, while the average emotional response scores increased linearly over the quarantine period. Similar to the findings of González-Sanguino et al. (21), the diverse components of stress in this study exhibited different patterns of change over time, which merits further investigation. However, days of continuous quarantine were not included in the final models of the total stress response, emotional response, and physical response. It is plausible that the effect of continuous quarantine time on individual stress responses existed but was weak, and the effect was covered up after other factors were taken into account. As one of the most detrimental factors to people's mental health (49), the impact of quarantine time on stress responses may be moderated by some other factors. In this study, we didn't observe any difference in perceived stress and stress responses at different quarantine locations, which contradicted the findings of a Poland study—Lipert, Musiał, and Rasmus reported that people who worked in workplaces had less stress than those who worked remotely (52), and a survey on Chinese quarantined people explaining that people in workplace had more social contact, which can help to relieve their stress (53). One possible explanation of our results is that by the third year of the pandemic, people have grown acclimated to life under the pandemic, so the different quarantine locations did not lead to significant differences in perceived stress and stress responses. Additionally, Chen and Sun stated that the perceived stress of those who were quarantined at centralized quarantine sites was significantly higher than that of those quarantined at home (54), whereas a global survey from 63 countries found an opposite result that people quarantined at home reported higher stress (13). The discrepancy may be due to cultural background or quarantine conditions. In our study, the subgroup at centralized quarantine sites was eliminated as the number of participants was <10. Further research is needed on differences in perceived stress and stress responses between centralized and non-centralized quarantines. In terms of the permitted scope of movement, it was observed that people who were not allowed to leave homes had the lowest perceived stress and stress responses. It can be explained that, compared with border scopes of movement, people confined to their homes have the lowest risk of infection, which may reduce their stress levels. Yet, the permitted scope of movement was not included in any of the final regression models.

Another factor worth discussing is occupations. No difference between occupations with and without the risk of COVID-19 exposure can be found in this research, which was inconsistent with most previous studies. The majority of studies have revealed that health care workers suffered significantly more stress than the general population (55, 56). And non-medical anti-epidemic workers in China were also found under huge stress and high exposure risks in the battle against the epidemic (57, 58). The inconsistent results can be explained from two aspects: On the one hand, anti-epidemic workers faced challenges such as inadequate virus knowledge, a shortage of personal protective equipment and work-overload at the beginning of the outbreak (59), which undoubtedly added to their stress. Now that these problems have been largely resolved, the psychological stress of anti-epidemic workers may be reduced; On the other hand, previous studies have found that front-line health workers reported significantly higher levels of stress than non-frontline workers and those who took on vacation during the pandemic (60–62). The participants with risky occupations in our study were all in quarantine, in that case, they may actually be less stressed than their colleagues at work, with no significant difference from the similarly quarantined public.

This study identified several risk factors for perceived stress and stress response during quarantine: youth, worries about personal finances and prolonged quarantine. These risk groups need more attention during the quarantine period. Governments are suggested to take measures to support those who have suffered financial losses as a result of quarantine (49), such as financial reimbursements, reducing corporate burdens by cutting or canceling interest rates on loans, deferment of taxes, keeping the payroll stable, and creating more jobs. It is also very important to minimize the duration of quarantine while providing adequate daily necessities for the quarantined people (49, 63), as well as releasing transparent, timely and accurate epidemic information (64). Although a great number mental health support services has already been introduced such as 24-h free psychological counseling hotline and free mental health assistance seminars (1), we still strongly recommend that mental health care systems and related organizations continue to provide various mental health assistance, as the impact of the quarantine on people may be long-lasting (49, 65). As for individuals during the quarantine, maintaining a normal routine and physical activity (9, 11, 66), doing some gardening (1), and reducing excessive attention to pandemic news (47) can be particularly helpful in alleviating psychological stress and promoting personal mental well-being.

## Limitation

There are several limitations of our study that should be noted in interpreting the results and explored in future research. Firstly, as with all cross-sectional designs and snowball sampling, this study had the limitations of not being able to make causal inferences and generalize the results to other situations. Secondly, we inferred lower levels of public stress in the third year of the pandemic compared to previous studies, but the comparison was cross-study and not by the same sample, so the inferences need to be interpreted with more caution. More reliable conclusions still need to be confirmed in longitudinal studies. Thirdly, some subgroups were under-representative (such as subgroups extremely worried about their finances and not permitted to leave their counties or districts) or were excluded (the subgroup at centralized quarantine sites). Further studies in larger samples should be conducted in the future. Last but not least, only a few factors that may be closely related to stress vulnerability were selected for this study based on previous studies. However, perceived stress and stress responses can be affected by more confounding factors. Some factors proven to reduce individuals' perceived stress, such as resilience and coping strategies (67, 68), may also have protective effects against stress responses, which require further investigation.

## Conclusion

During the third year of the COVID-19 pandemic, the perceived stress and stress responses of the Chinese quarantined population were generally stable. People's stress levels were associated with youth, higher levels of financial worries, and longer periods of quarantine, with financial worries being the main determinant of people's perceived stress and various stress responses. Therefore, we recommend that non-essential periods of quarantine be minimized, and that changes in the people's psychological status be continuously monitored during the quarantine period, so as to provide timely and effective mental health services to the quarantined people. Meanwhile, relevant authorities should pay more attention to risk groups, especially those worried about their financial situations, and provide them with additional support and assistance if necessary.

## Data availability statement

The raw data supporting the conclusions of this article will be made available by the authors, without undue reservation.

## Ethics statement

The studies involving human participants were reviewed and approved by the Ethics Committee of Naval Medical University. The patients/participants provided their written informed consent to participate in this study.

## Author contributions

QG: study design, data analysis, and drafting of the manuscript. KT: data analysis. HJ, CZ, QJ, and XL: data collection. YZ: critical revision. TS: study design, critical revision. All authors contributed to the article and approved the submitted version.

## Funding

This study was funded by Pyramid Talents Project-Military medical talents project of Changzheng Hospital (202007A02), Naval Military Theoretical Research Program in 2020 (202058-80), Construction Project of Key Disciplines and Specialties of 13th 5-Year Plan of PLA (2020SZ29).

## Conflict of interest

The authors declare that the research was conducted in the absence of any commercial or financial relationships that could be construed as a potential conflict of interest.

## Publisher's note

All claims expressed in this article are solely those of the authors and do not necessarily represent those of their affiliated organizations, or those of the publisher, the editors and the reviewers. Any product that may be evaluated in this article, or claim that may be made by its manufacturer, is not guaranteed or endorsed by the publisher.
